# Super-resolution imaging using nano-bells

**DOI:** 10.1038/s41598-018-34744-6

**Published:** 2018-11-06

**Authors:** Rafael Fuentes-Domínguez, Fernando Pérez-Cota, Shakila Naznin, Richard J. Smith, Matt Clark

**Affiliations:** Optics and Photonics Group, University of Nottingham, University Park, Nottingham, NG7 2RD UK

## Abstract

In this paper we demonstrate a new scheme for optical super-resolution, inspired, in-part, by PALM and STORM. In this scheme each object in the field of view is tagged with a signal that allows them to be detected separately. By doing this we can identify and locate each object separately with significantly higher resolution than the diffraction limit. We demonstrate this by imaging nanoparticles significantly smaller than the optical resolution limit. In this case the “tag” we have used is the frequency of vibration of nanoscale “bells” made of metallic nanoparticles whose acoustic vibrational frequency is in the multi-GHz range. Since the vibration of the particles can be easily excited and detected and the frequency is directly related to the particle size, we can separate the signals from many particles of sufficiently different sizes even though they are smaller than, and separated by less than, the optical resolution limit. Using this scheme we have been able to localise the nanoparticle position with a precision of ~3 nm. This has many potential advantages - such nanoparticles are easily inserted into cells and well tolerated, the particles do not bleach and can be produced easily with very dispersed sizes. We estimate that 50 or more different particles (or frequency channels) can be accessed in each optical point spread function using the vibrational frequencies of gold nanospheres. However, many more channels may be accessed using more complex structures (such as nanorods) and detection techniques (for instance using polarization or wavelength selective detection) opening up this technique as a generalized method of achieving super-optical resolution imaging.

## Introduction

Conventional optical imaging is restricted in resolution by the Rayleigh criterion to ~*λ*_optical_/NA, where *λ*_optical_ is the optical wavelength and NA is the numerical aperture^1^ of the objective lens. Even for sophisticated immersion lens microscopes the NA is limited to <1.5. This practically limits conventional optical microscopy resolution to a few hundred nanometres.

There is significant interest in imaging at higher resolutions than this limit permits, especially in biology, where, until recently, optical microscopy has been the only technique available for imaging live cells in this way^2,3^. As such optical microscopy is one of the most important tools in the life sciences because other techniques, such as electron microscopy can only operate on dead cells and tissue.

Higher resolution can be achieved using near field or super resolution techniques such as stimulated emission depletion (STED)^4^ microscopy or photo activated localisation (PALM) and stochastic optical reconstruction (STORM) microscopy^5,6^. In these schemes fluorophores and switchable fluorophores are required to enable super resolution along with high photon dose at short optical wavelengths which are used to pump and switch the fluorophores. STED works by optically “turning off” the fluorophores around the imaging point using a ring-shape pattern of light leaving a smaller (than the optical resolution limit) active area which is then detected. PALM and STORM rely on switchable fluorophores which blink on and off between photoactive and dark states. By observing the blinking, the optical point spread function (PSF) at the detector can be attributed to individual fluorophores and these fluorophores can then be super-localised (localised with greater precision that the optical resolution). These techniques have produced important results, however they require typically high light intensities which produce photodamage and they bleach. This is particularly important for dynamic studies where repeated imaging of a specimens such as cells is required and results in cell death or loss of the capability for super-resolution.

Metal nanoparticles have been used previously in optical super-resolution microscopy to enhance or substitute fluorophores. Core-shell nanoparticles, consisting of a dye-doped silica core covered with a layer of gold, were used in STED and a reduction of the STED depletion power was demonstrated due to the field enhancement provided by the localized surface plasmon resonance (LSPR)^7^. The change in the medium refractive index due to the local heat rise when a metal nanoparticle is excited near the LSPR has been used by photothermal imaging to detect gold colloids down to diameter of 2.5 nm^8^. In addition to this, a wide-field interferometric phase microscopy has been implemented to create a map of the locations of gold nanoparticles in a sample without the need of scanning^9^.

In this paper we demonstrate that nanoparticles can be super-localised by exploiting their acoustic resonances. We propose to use this feature to achieve super-optical resolution. This is a powerful concept because it opens up the possibility of unbleacheable labels, multiplexing many super-optical resolution imaging schemes *and* a route to contrast derived by elasticity at the super-resolution level.

## Nano-bells

Nanoparticle vibrations have been extensively studied by a variety of methods, especially with time resolved optical pump-probe techniques^10–14^ where their vibrational frequencies have been well characterised with modelling and experimental data. Inspired by this and localisation microscopy^5,6^, we propose “nano-bells”. This is a completely novel tagging method for localisation/super-resolution microscopy.

**Figure 1:**
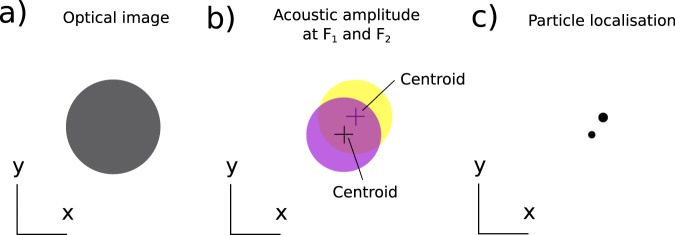
How nano-bells work. (**a**) Shows a representation of an optical image of metallic particles where the particles can not be resolved. (**b**) Shows a representation of an acoustic amplitude image of the same particles at a single frequency F_1_ and F_2_. (**c**) Localisation of the particles from the centroids of the PSF of each frequency. The centroids obtained at frequencies F_1_ and F_2_ lead to the localisation of the particles with greater precision than the optical system used to image them. The size is obtained by the vibrational frequency of each particle.

In “nano-bells” (see Fig. 1), the tags are metallic nanoparticles where their optical PSF overlap in time and space and hence can not be resolved. However, it is possible to separate the PSF of each particle by measuring their vibrational signatures provided the particles are of different size and hence vibrate at a different frequency (see Fig. 1b). Determining the centroid of the point-spread function for each frequency, super-localisation can be achieved (see Fig. 1c). In “nano-bells”, we propose to use the acoustic characteristics of nanoparticles as a route for super-resolution reconstruction imaging in a similar way to STORM^15^.

## Results

### Modelling and characterisation of nano-bells

Not all metallic particles are suitable for nano-bells. Gold nanoparticles were chosen to facilitate the scheme, in this case they were required to strongly absorb the pump beam (420 nm), strongly scatter and exhibit a large change of scattering cross section with change of size at the probe beam wavelength (780 nm) and have a fundamental breathing mode frequency in the measurement range (80 MHz–1THz). Neither wavelength used is critical to the final precision achieved.

**Figure 2:**
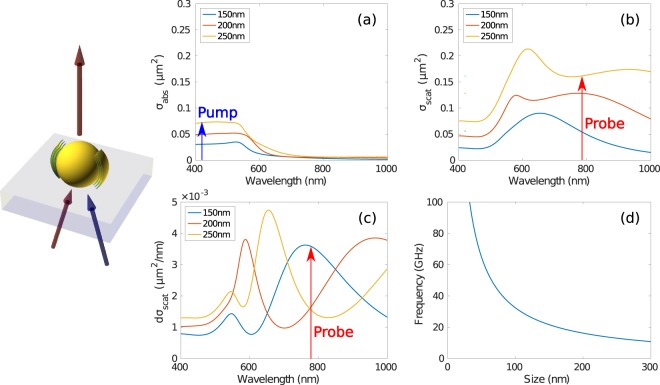
Modelled response of nano-bells. (left) Schematic of the experiment from the nanoparticles’ point of view. The particle sits on the transparent substrate and is illuminated by the pump beam and probe beam from below. The scattered light is collect in transmission from above. (**a**) The optical absorption cross section for the gold nanoparticles used in the experiment calculated using Mie theory for the pump wavelength. (**b**) The optical scattering cross section calculated for the probe wavelength, (**c**) the derivative of (**b**) with respect to size, and (**d**) the vibration frequencies of the particles as a function of size.

Figure 2 shows a schematic of the experiment from the particle’s point of view; the absorption cross section and scattering cross section as a function wavelength with the pump and probe wavelengths marked for three particle sizes 150–250 nm; the derivative of the scattering cross section with respect to particle size as a function of wavelength; and the breathing mode frequency as a function of size. In this scheme the pump excites the fundamental breathing mode of vibration and the vibration modulates the size of the particle which modulates the scattering cross section at the probe wavelength^16^. Using Fig. 2(d) and an instrument bandwidth range of 10–80 GHz it can be seen that the particles in the size range 300 nm to 30 nm can be used in this configuration.

### Experimental measurement of nanoparticle vibrations

Substrates with a variety of gold nanospheres were fabricated by drop casting solutions of various nanoparticle concentrations on to gridded coverslips (see methods). Gridded coverslips were used to facilitate the location of the nanoparticles in our experimental system and during scanning electron microscopy (SEM) to ensure the same nanoparticles were observed and measured in each system. SEM imaging of the particles was performed before and after acoustic measurements which confirmed that there was no damage or change to the samples.

The experimental system consisted of an inverted microscope with additional elements mounted above the optical deck. The sample was mounted on a positioning stage with 100 nm step size for raster scanning. Laser illumination was delivered from below using a x20 objective lens and the scattered probe light was collected above using an additional x20 objective (see methods).

**Figure 3:**
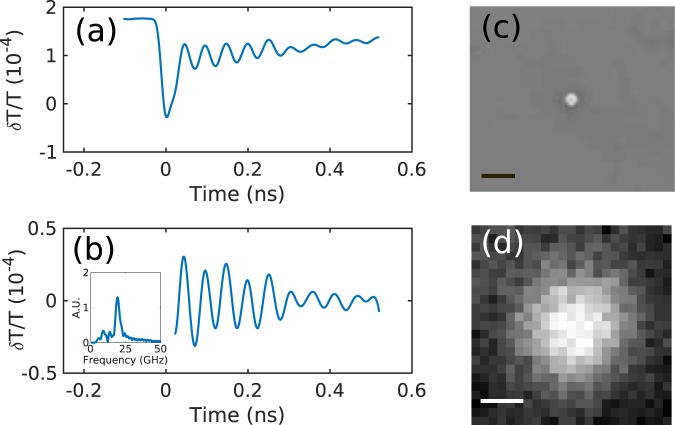
(**a**) Raw signal centred on the particle, (**b**) signal after processing to extract the change in scattering cross section as the particle vibrates (inset frequency content), (**c**) SEM of the particle and (**d**) optical image of same particle. Scale bars in (**c**) and (**d**) 500 nm.

Figure 3 shows a typical signal taken from the system: (a) raw signal taken centred on the particle, (b) signal after processing to extract the change due to scattering cross section modulation as the particle vibrates (inset frequency content), (c) SEM of the particle and (d) optical image of same particle. We can use the frequency of vibration to determine the size of the particle, in this case the size is estimated as 168 ± 2 nm from a 18.9 GHz breathing mode.

### Super-localisation of multiple nano-bells

**Figure 4:**
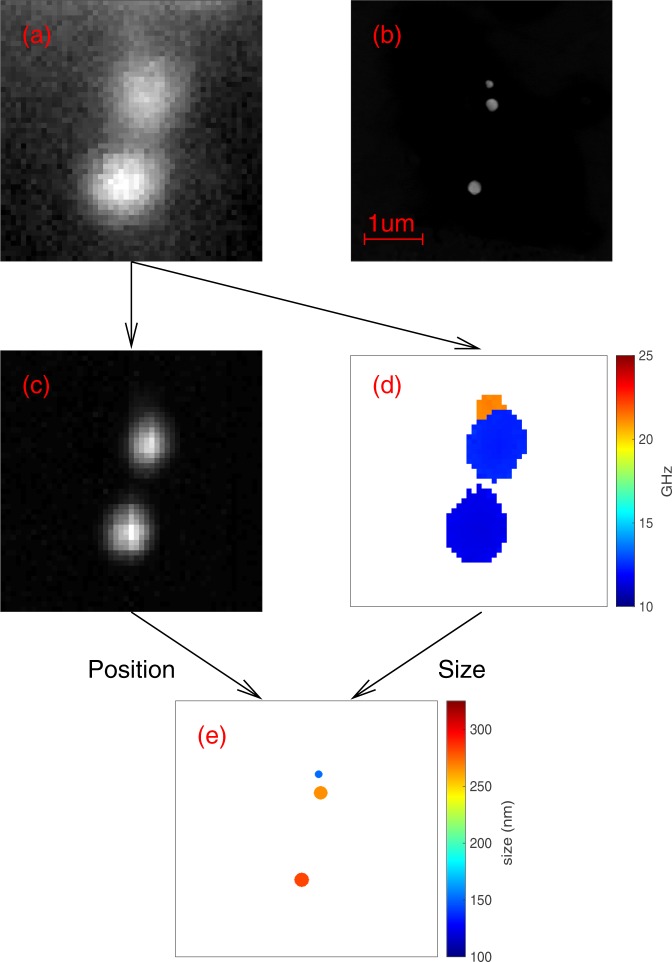
Images of three particles. (**a**) Optical image, (**b**) SEM, (**c**) acoustic amplitude, (**d**) acoustic frequency and (**e**) localisation of the particles with better precision than the diffraction limit. One is well separated from the others and can easily be resolved optically. The other two are ~200 nm apart and cannot be resolved optically, however, the acoustic signals clearly identifies the size and location of the particles allowing accurate reconstruction of the image shown in the SEM.

Figure 4 shows a sequence of images taken using our GHz/nanoscale phononic microscope (see methods) of three particles deposited on a gridded coverslip, (a) is the optical image, (b) the SEM image of the same area, (c) the amplitude of the vibrational signal, (d) the acoustic frequency measured from the dominant peak and (e) the reconstruction of the image using (c) to locate the particles and (d) to determine their size.

In Fig. 4a the picture shows two objects well separated and because of this, one can assume their localisation is trivial by optical means. However, the SEM image of the same area (see Fig. 4b) reveals a third object not localised by optical imaging. In this case two of the three particles are close together, separated by less than the width of the optical PSF so the reconstruction becomes more complex.

Here we rely on the vibrational frequency of nano-bells to attribute, like in STORM, a unique point-spread function to each particle. The acoustic scan provides the amplitude map of the particles which still shows only two objects (see Fig. 4c). However plotting the amplitude of each frequency present in the detected signal, lends to the reconstruction of the PSF of each particle (see Fig. 4d). Once the PSF of each particle is detected using its frequency signature, its location is determined by centroiding its PSF in a similar way to STORM (see Fig. 4e). Note that the PSF of different particles overlap although is not visible in Fig. 4d.

It can be seen that the ultrasonic reconstruction has successfully localised the nanoparticles in the sample at far higher precision than the resolution of the optical microscope is capable of. The precision in which the size and position of the particles are localised, determined from the signal and noise analysis, can be measured to ~3 nm (see methods). It is important to note that (with the exception of the SEM images) all the images presented in this paper were made through the same optics and all are constrained by the same conventional optical resolution.

**Figure 5:**
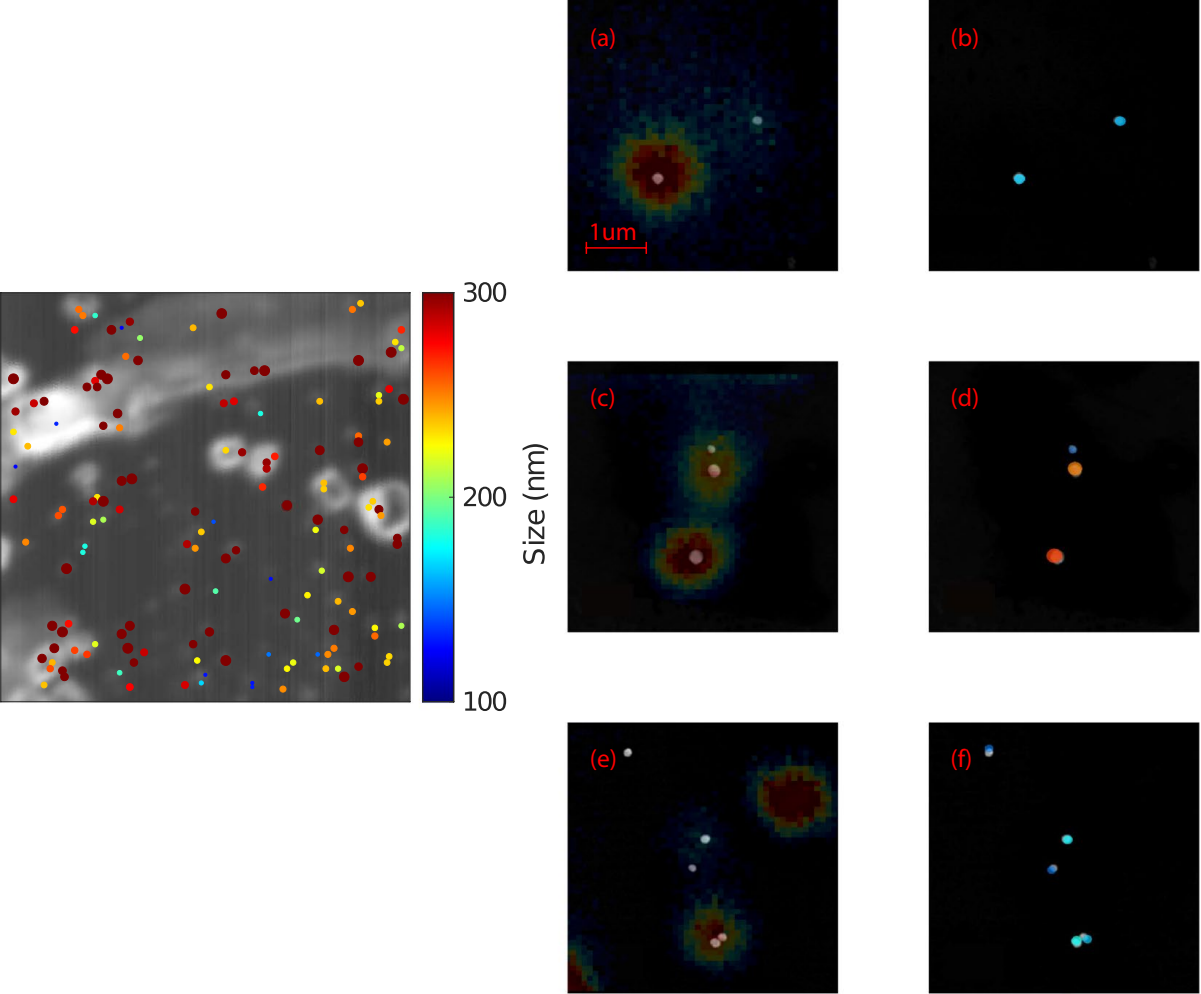
(Left) 50 × 50 *μ*m area image showing ~150 nanoparticles and their predicted sizes where the particles are shown at 300% of their measured size for clarity at this scale. (Right) (**a**,**c**,**e**) SEMs of nanoparticles overlaid with their optical images and (**b**,**d**,**f**) SEMs of the nanoparticles shown in (**a**,**c**,**e**) overlaid with the acoustic reconstruction where super-optical resolution is shown. False colour in (**b**,**d**,**f**) indicates particle size on the same color scale as the left figure.

This process breaks down in the event of multiple particles with the same frequency of vibration being imaged in the same optical point spread function similar to when two fluorophores turn on simultaneously within one PSF in STORM. However, if the particles in each optical point spread function are sufficiently size dispersed then they can be easily and reliably imaged. Figure 5 shows a large scale image identifying many particles in a 50 × 50 *μ*m field of view and super-resolution images for three different positions on our sample demonstrating reliable and robust reconstruction.

## Discussion

By exploiting the polydispersity of the nanoparticles, and the different vibrational frequencies they exhibit, we have been able to determine their location and size at precision far higher than the native optical resolution of our system. We assume (a priori) that the particle is a sphere. For more complex particles, such as nanorods, it can be possible to also determine the shape, critical dimensions and orientation from their acoustic signatures but this is beyond this paper.

Metallic particles are easily functionalised and well tolerated by biological cells^17–19^. We propose then to tag cells with “nano-bells” for super-resolution reconstruction imaging in the same way that fluorophores are funcionalised and used in other super-resolution techniques. This new scheme for super-resolution imaging may offer significant advantages for living-cell applications over techniques based on fluorescent dyes. Nanoparticles will not bleach allowing long term repeated imaging. The phonons will not cause damage even though they have sub-optical wavelengths. Particles can be accessed and detected through relatively long optical wavelengths reducing phototoxicity and the vibrational modes of particles offer the possibility of mechanical characterisation^2,11^.

Nano-bells offers potential advantages for cell-imaging, however substantial technological challenges must be addressed. First, acquisition speed is not yet practical for biological applications and it could be increased through for instance wide-field detection. Second, current particle sizes are relatively large compared to the PSF of the optical system, however these were selected for ease of use and much smaller particles can be used if the optical probing wavelength is reduced. Third and final, biocompatibility of the current scheme might be low due to absorption of the excitation wavelength (420 nm) and generation of heat. Selecting an adequate material, a longer wavelength can be used for excitation and the thermal load which is very small for a single pulse can be reduced if the acquisition speed and signal-to-noise ratio increase. We expect that future technological developments will allow nano-bells to be a competitive super-resolution alternative.

In a normal optical imaging system the resolution is determined by considering when adjacent optical point spread functions overlap and can no longer be distinguished. In super-resolution localisation microscopy a different criteria is considered and that is the precision with which a single fluorophore can be localised. In the scheme presented in this paper a similar approach is used and the resolution should become the precision of the localisation of the particle and, additionally, if required, consideration of the precision of the particle sizing.

Analysis of the signals and noise in the system puts the localisation precision for the images shown in this paper to be ~3 nm and the size accuracy to be ~2 nm (see methods). However, these noise derived figures need to be treated carefully–while the measurements are repeatable to these errors, the assumptions that we have used to derive the images are slightly naive. For instance, we treat the vibration of the particles and optical scattering from the particles as if they were suspended in free space and completely isolated from any optical or mechanical influences. The reality of the situation is that the particles are sat on a hard elastic surface and the optical refractive index of the surface differs from that of air. However, this does not appear to affect our ability to super-localise the particles.

In normal optical imaging the axial resolution is determined by the axial point spread function and normally given by ~*λ*/NA^2^. In this paper the particles all line on the plane of the substrate and axial resolution is not considered. However, the axial resolution of our system in a three dimensional sample would be determined by one of two schemes: firstly the standard optical resolution determined by the optical axial point spread function and secondly if the system were able to pick up the acoustic waves on the substrate then the axial resolution would be determined by the acoustic wavelength which has already been demonstrated to be better than 200 nm^2^.

In addition to the usual limits on resolution described above there is an additional consideration: the number of localised particles or *channels* available. In the examples shown in this paper six different particles sizes were used that would result in a maximum of six localised particles per optical point spread function. In a sample with a random mix of particles there is a large probability that two particles of the same size will be within one point spread function and this will result in these particles being unlocalised.

The total number of channels available is a function of the particle properties and the instrumentation. In this case, the frequency bandwidth of the vibration is determined by the vibrational decay of the particles due to thermal relaxation, phonon scattering and radiation of acoustic waves into the medium around the particles. The bandwidth of the instrumentation is potentially very high but the optical detection method (size modulation of the scattering) restricts the size range of the particles that can be detected (although alternative methods^20^ go much higher in frequency).

For our implementation the practical number of channels is estimated to be around 50 (see methods). For a random arrangement of particles with 50 available channels, we estimate that 7 particles in a PSF will have less than 50% probability of having unlocalised particles (two or more particles of the same size). This can be dramatically increased using a more complex particle, for instance nanorods^21^ or nanoshells^22^ because these exhibit richer acoustic behaviour and more particles can be detected with orthogonal signals. It must be noted that increasing the number of channels decreases the probability of two particles of the same size occurring in the same PSF. However this does not increase the number of channels that can be measured from a single PSF simultaneously. Instead, this number is largely limited by the physical dimensions of the particles and the object that they are intending to image.

When two or more particles are very close or touching the situation becomes more complex still. Close but not mechanically coupled particles can still have complex coupled electromagnetic resonances and these can result in high sensitivity to certain vibrational modes which can be detected optically^23^. In addition situations where there is physical contact, extra vibrational modes and signals can exist that may also be detected. This can be a rich source of additional information but can be very complex and difficult to invert to determine the image. The coupling to the matrix or substrate can also lead to additional frequency components in the signals which tends to lie at lower frequencies than the main vibrational modes that we observe^24^. These signals can provide additional information about the coupling of the particles to the substrate and media. In finite element modelling studies we were able to observe that there was no significant problems separating signals from different particles when they were further than 10–20 nm apart.

We have shown how the vibrational frequencies of different size nanoparticles can be used to isolate and pick out the optical signal from individual particles even if they are imaged together within the same optical point-spread function. We proposed to use “nano-bells” as a novel route to imaging with super-optical resolution. This potential new technique can be implemented through a conventional optical microscope, remove the limitations of bleaching, be live-cell compatible^18,19^ and lead to super-resolved mechanical characterisation.

## Methods

### Substrate preparation

A lithography process was used to make the gridded cover-slips. A photoresist coating (BPRS 150) was coated on a clean cover-slip and then, the ultraviolet (UV) exposure was performed using a Karl Suss MJB3 Mask Aligner with a power of 7 mW/cm^2^ through the gridded mask. Later, a developing solution (1:8) of AZ400K and deionised (DI) water was used for the developing process, and the cover-slip was washed with DI water and dried with nitrogen gas. Then, 10 nm of indium tin oxide (ITO) and 20 nm of gold (Au) were coated and lift-off process was made using warm acetone in an ultrasonic bath. Finally a thin ITO film, ~50 nm, was sputter coated over the entire surface before depositing the gold nanoparticles on the gridded cover-slip. This extra ITO layer avoids charging effects during scanning electron microscopy (SEM) so that reference images could be taken of the particles. The gridded cover slip contained reference coordinates that were clearly visible in both optical and SEM microscopes so that the same imaging area and orientation could easily be found in all instruments.

### Nanoparticle deposition

The gold nanoparticles used in this sample preparation were purchased from Sigma-Aldrich, UK. The nanoparticles were used as received with no surface modification or washing step involved. Their polydispersity index (PDI) were less than 20% (values provided by manufacturers) so six different size batches on nanoparticles were mixed to increase the size dispersity. The nominal particle sizes were 80, 100, 125, 150, 200 and 250 nm. The nanoparticles were suspended in water, drop coated onto the gridded substrates and then dried overnight.

### Experimental setup

**Figure 6:**
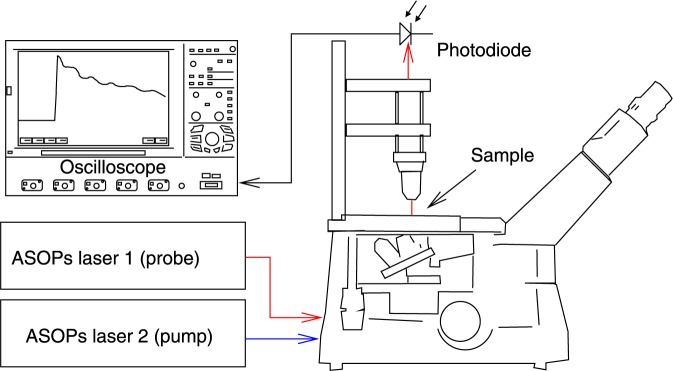
Schematic of the super-resolving phononic microscope.

The imaging experiment was conducted using a custom microscope built around an Olympus IX73 inverted microscope body (Fig. 6). This was arranged in a conventional inverted microscope layout with and additional object lens on top, similar to a 4*π* microscope layout. With this arrangement it was possible to pump and probe and image the particles from one or both sides of the sample simultaneously. In addition to conventional imaging optics the system allowed two laser beams to be focused into the sample. These beams were provided by a dual Ti:Sapphire (Tsunami, Spectra-Physics) laser asynchronous optical sampling system (ASOPS)^25,26^ and produces ~100 fs pulses with an 80 MHz repetition rate. The ASOPS electronics allow the timing of the laser pulse from each laser to be precisely controlled and for the time delay between the pulses to be swept from 0–12.5 ns every 100 *μ*s (10 kHz). While the lasers were wavelength tunable, these experiments were performed with fixed wavelengths of 420 nm and 780 nm for the pump and probe beams respectively. They were brought to the sample focused an Olympus LUC PLFLN x20 objective (0.45NA). This produced an optical point spread function of ~1 *μ*m (FWHM) which limits the optical resolution to around 1 *μ*m.

Maximum average powers of 0.5 mW pump and 1 mW probe (measured at sample) were used, corresponding to pulse energies of 6.25pJ and 12.5pJ and peak powers of 62.5 W and 125 W, respectively. The sample was scanned by moving electromechanical stages (Thorlabs MLS 203–1) with a minimum step motion of 100 nm. The probe beam was detected using a Thorlabs PDA36A which effective low pass filters the optical signal removing the individual pulses. The signal was then collected by a Lecroy HRO66Zi oscilloscope as the ASOPS delay was swept from 0–12.5 ns. With this arrangement optical frequencies detected were down shifted by a factor of 8000 with the 0–100 GHz frequencies being converted to 0–12.5 MHz. The oscilloscope performed high speed averaging of this signal and, typically, 30,000 averages per point are taken during scanning which takes around 9 s to acquire.

### Signal processing

The raw signal went through several stages of processing to extract the vibration amplitude and frequencies. The individual laser pulses (100 fs temporal pulse length, 80 MHz repetition rate) are time averaged on the optical detector which had a bandwidth of 10 MHz. The detected signal contained several components: coincidence peak, thermal response and oscillation signal (see Fig. 3(a)). The coincidence peak and thermal response are removed by curve fitting leaving a decaying oscillatory component caused by the elastic vibration of the particles (Fig. 3(b)). A fast Fourier transform (FFT) is then performed on the resultant trace to measure the main vibrational mode of the nanoparticles. Once the Fourier components have been computed any particles present can be detected as frequency peak in the spectrum. The size of the particles can be computed using Fig. 2(d). The particle can be localised by centroiding the amplitude at its vibration frequency (Fig. 4(c)).

### Image reconstruction and localisation

Once the data was signal processed (above) the image was reconstructed by identifying peaks in the Fourier spectra caused by vibrating particles. The frequency of vibration was used to identify the size of the particle and then the spatial distribution of the vibration amplitude at that frequency (a vibration point spread function) was centroided to determine the particles’ location. A two stage centroiding algorithm was employed to reduce the contribution of noise from outside of the point spread function, first an initial centroid was computed using all the data, then a Gaussian mask (width approximately two optical point spread functions wide and not critical) was applied to suppress noise from outside of the PSF and the centroid was recomputed.

### SEM imaging

Prior to and after optical imaging the samples were imaged using a JEOL 7100F FEG-SEM electron microscope using a voltage of 15 keV, x19,000 magnification for the high resolution images and a working distance of 10 mm. The gridded coverslips were overcoated with 50 nm of ITO prior to particle deposition in order to remove artefacts caused by charge build up. This step avoids the need of coating the whole sample with a metal layer (after deposition the particles) which will affect the acoustic data and the vibrational modes of the nano-bells.

### Image registration and alignment

The imaging areas in the SEM and optical microscope were registered using the indexed grid lines fabricated on the coverslips which could be clearly observed in both instruments at low magnification. The grid lines were used to rotate the optical images to match the SEM, the relative scaling of the SEM and optical images was performed using the scale bars in the SEM images and the positional information from the microscope stage respectively. Initial registration of the images was performed using the grid lines, however, final registration was performed by eye in Fig. 4 because the grid lines were not visible in the high resolution SEM images.

### Noise and localisation error estimation

The noise contribution to the positional error in the centroiding was estimated by measuring the standard deviation of signal level in regions with no particles and using this as a measure of the signal noise. This error was then used with simple error propagation analysis to estimate the position error of the calculated centroids and these calculations were confirmed by simulation of signals with random noise added. This gave an estimation of the positional error for our system as 3 nm for the typical signals shown in the results. This was achieved by manually identifying a rough window location for each particle, centroiding to get a first estimate of the particles and using this estimate to position a Gaussian mask (approximately twice optical point spread function size) to suppress noise from dark pixels outside the particle position. The particle size error was estimated by considering the effect of background noise on the measurement.

### Channel width and number estimation and spatial bandwidth determination

This technique requires that individual particles can be differentiated. The number of different particles (or “channels”) that the system can observe simultaneously determines the ultimate spatial-bandwidth of the system when imaging arbitrary objects. When imaging typical objects the volume occupied by high contrast objects is usually a small fraction of the total imaging volume in which case the imaging resolution is determined by the spatial localisation accuracy rather than the number of channels available. In the case of the nanospheres resonances used in this paper we detect a single vibrational frequency for each particle: the breathing mode frequency. This is because of a combination of factors; the breathing mode is preferentially excited by the pump beam (because it is effectively uniformly heated due to the action of hot electrons rapidly spread heat^27^) and the detection mechanism is most sensitive to the breathing mode size changes. The number of channels can therefore be determined simply by considering the frequency separation required to localise two particles (~1.5 GHz) within the system bandwidth (1THz). This gives an indication of the maximum number of different particles that could be imaged separately in the volume or area of one optical point spread function (~50 different particles). If we consider a more complex particle it would be possible to detect additional non-redundant vibration frequencies and multiplex additional signal using wavelength or polarisation tagging and the number of channels could be increased considerably.
